# Testing non-inferiority of a new treatment in three-arm clinical trials with binary endpoints

**DOI:** 10.1186/1471-2288-14-134

**Published:** 2014-12-18

**Authors:** Nian-Sheng Tang, Bin Yu, Man-Lai Tang

**Affiliations:** Department of Statistics, Yunnan University, No.2 Cuihu North Road, 650091 Kunming, China; Department of Mathematics and Statistics, Hang Seng Management College, Hang Seng Link, Siu Lek Yuen, Shatin NT, Hong Kong, China

**Keywords:** Approximate unconditional test, Bootstrap-resampling test, Non-inferiority trial, Rate difference, Saddlepoint approximation, Three-arm design

## Abstract

**Background:**

A two-arm non-inferiority trial without a placebo is usually adopted to demonstrate that an experimental treatment is not worse than a reference treatment by a small pre-specified *non-inferiority margin* due to ethical concerns. *Selection of the non-inferiority margin* and *establishment of assay sensitivity* are two major issues in the design, analysis and interpretation for two-arm non-inferiority trials. Alternatively, a three-arm non-inferiority clinical trial including a placebo is usually conducted to assess the assay sensitivity and internal validity of a trial. Recently, some large-sample approaches have been developed to assess the non-inferiority of a new treatment based on the three-arm trial design. However, these methods behave badly with small sample sizes in the three arms. This manuscript aims to develop some reliable small-sample methods to test three-arm non-inferiority.

**Methods:**

Saddlepoint approximation, exact and approximate unconditional, and bootstrap-resampling methods are developed to calculate p-values of the Wald-type, score and likelihood ratio tests. Simulation studies are conducted to evaluate their performance in terms of type I error rate and power.

**Results:**

Our empirical results show that the saddlepoint approximation method generally behaves better than the asymptotic method based on the Wald-type test statistic. For small sample sizes, approximate unconditional and bootstrap-resampling methods based on the score test statistic perform better in the sense that their corresponding type I error rates are generally closer to the prespecified nominal level than those of other test procedures.

**Conclusions:**

Both approximate unconditional and bootstrap-resampling test procedures based on the score test statistic are generally recommended for three-arm non-inferiority trials with binary outcomes.

## Background

The objective of a non-inferiority trial is to demonstrate the efficacy of an experimental treatment not being inferior to a reference treatment by some pre-specified non-inferiority margin. Many authors considered two-arm non-inferiority trials without a placebo since the comparison between the experimental and reference treatments is direct and the potential ethical problems encountered in traditional placebo-controlled trials are avoided (for example, see Dunnett and Gent [1], Tango [2], and Tang et al. [3]). However, there are two major concerns for two-arm non-inferiority trials [4]. The first issue is the choice of the non-inferiority margin, which is the clinically acceptable amount or a combination of statistical reasoning and clinical judgement. The other issue is the evaluation of assay sensitivity, which refers to the ability of a trial to differentiate an effective treatment from a less effective or ineffective treatment [5]. Without a placebo arm, the assay sensitivity of a trail is not demonstrable from the trial data and ones must rely on some external information (e.g., historical placebo trails) for the reference treatment [4]. Without the trial assay sensitivity, any non-inferiority testing results from the comparison of the experimental and reference treatments will become unconvincing. There are some indications where it is considered ethically acceptable to continue to randomize patients to placebo despite the fact that an effective treatment exists and there is interest in seeing not only whether the new treatment works at all but also how it measures up to accepted therapy. In this case, a three-arm non-inferiority clinical trail including the experimental treatment, an active reference treatment and a placebo is usually conducted to assess assay sensitivity and internal validation of a trail [6]. Indeed, three-arm trials are recommended in the guidelines of the ICH (The International Conference on Harmonisation of Technical Requirements for Registration of Pharmaceuticals for Human Use) and EMEA/CPMP (European Medicines Agency/Committee for Proprietary Medical Products) as a useful approach to the assessment of assay sensitivity and internal validation (e.g., see [7]).

Statistical inference based on three-arm non-inferiority clinical trials with normally distributed outcomes has received considerable attention in recent years. For example, Koch and Tangen [8] and Pigeot et al. [9] considered the problem of three-arm non-inferiority testing for normally distributed endpoints with a common but unknown variance. Koti [10] presented a new approach for normally distributed endpoints based on the Fieller-Hinkley distribution. Hasler, Vonk and Hothorn [11] proposed the usage of the *t*-distribution in the presence of heteroscedasticity. Hida and Tango [7] proposed a test procedure for assessing the assay sensitivity with a pre-specified margin defined as a difference between treatments in the presence of homoscedasticity. Ghosh, Nathoo, Gönen and Tiwari [12] developed a Bayesian approach in the presence of heteroscedasticity by incorporating both parametric and semi-parametric models. Gamalo, Muthukumarana, Ghosh and Tiwari [13] extended the existing generalized p-value approach for assessing the non-inferiority of a new treatment in a three-arm trial.

Recently, some statistical methods have also been developed for three-arm non-inferiority testing with binary endpoints. For example, Tang and Tang [14] proposed two asymptotic approaches for testing three-arm non-inferiority via rate difference based on Wald-type and score test statistics. Kieser and Friede (2007) revisited the performance of Tang and Tang’s [14] asymptotic test statistics via simulation studies and derived approximate sample size formulae for achieving the desired power. Munk, Mielke, Skipka and Freitag [15] developed likelihood ratio tests. Li and Gao [4] used the closed testing principle to establish the hierarchical testing procedure and proposed a group sequential type design. Liu, Tzeng and Tsou [16] presented a three-step testing procedure and derived an optimal sample size allocation rule in an ethical and reliable manner that minimizes the total sample size.

All aforementioned approaches for testing non-inferiority of a new treatment in a three-arm clinical trial with binary endpoints are based on large sample theory, and their accuracy has long been suspected and criticized when sample sizes are small or the data structure is sparse. To the best of our knowledge, limited work have been done to address these issues. Motivated by Jensen [17], we derive saddlepoint approximations to the cumulative distribution functions of Wald-type, score and likelihood ratio test statistics. Inspired by Tang and Tang [18], we also propose the exact unconditional, approximate unconditional and Bootstrap-resampling *p*-value calculation procedures for testing three-arm non-inferiority with small sample sizes.

The rest of this article is organized as follows. We first review three test statistics for assessing non-inferiority of a new treatment in three-arm clinical trials with binary endpoints. We also propose saddlepoint approximation, exact and approximate unconditional, and bootstrap-resampling approaches for calculating *p*-values. Simulation studies are conducted to investigate the performance of all test statistics based on different *p*-value calculation approaches in terms of type I error rate and power. An example is analyzed to demonstrate our methodologies. Finally, we discuss the performance of our proposed methodologies and present some conclusions.

## Methods

### Model

Let consider a clinical trial with the test (T), reference (R) and placebo (P) treatments, and assume their primary clinical outcomes *X*_*T*_, *X*_*R*_ and *X*_*P*_ be independent and binomially distributed as *X*_*T*_∼Bin(*n*_*T*_,*π*_*T*_), *X*_*R*_∼Bin(*n*_*R*_,*π*_*R*_) and *X*_*P*_∼Bin(*n*_*P*_,*π*_*P*_), respectively. Here, *X*_*T*_,*X*_*R*_ and *X*_*P*_ are the numbers of responses in groups T, R and P, respectively, *π*_*T*_,*π*_*R*_ and *π*_*P*_ represent their corresponding response probabilities with higher probability indicating a more favorable outcome, and *n*_*T*_,*n*_*R*_ and *n*_*P*_ denote their corresponding sample sizes. Thus, the joint probability density function of (*x*_*T*_,*x*_*R*_,*x*_*P*_) is given by
2.1

It can be easily shown from Equation (2.1) that the maximum likelihood estimates (MLEs) of *π*_*T*_, *π*_*R*_ and *π*_*P*_ are given by ,  and , respectively.

### Test statistics

Following Hida and Tango [7], to test the non-inferiority of the experimental treatment to the reference with the assay sensitivity in a three-arm trial, we have to simultaneously demonstrate (i) the superiority of the experimental treatment to the placebo, (ii) the non-inferiority of the experimental treatment to the reference with a non-inferiority margin *Δ*>0, and (iii) the superiority of the reference treatment to the placebo by more than *Δ*. That is, *π*_*T*_, *π*_*R*_ and *π*_*P*_ must satisfy the following inequalities: *π*_*P*_<*π*_*R*_−*Δ*<*π*_*T*_, which can be written as the following two hypotheses:


Similar to Pigeot et al. [9], we take the margin *Δ* as a fraction *f* of the effect size of the reference treatment, i.e., *Δ*=*f*(*π*_*R*_−*π*_*P*_). Generally, one can select *f*=1/2 and 1/3 [14]. Thus, the second hypothesis can be expressed as *K*_0_:*π*_*R*_≤*π*_*P*_ versus *K*_1_:*π*_*R*_>*π*_*P*_. If *K*_0_ is rejected, letting *f*=1−*θ* yields the following non-inferiority hypothesis:
2.2

where *θ*∈(0,1) is a fixed retention fraction [8]. Rejecting *H*_0_ implies that the test treatment preserves at least 100*θ%* of the efficacy of the reference treatment compared to placebo [19]. Similar to Tang and Tang [14], we only consider hypothesis *H*_0_ and assume that *K*_0_ is rejected at some pre-given significant level. Thus, the non-inferiority hypothesis (2.2) can be rewritten as
2.3

Let *ψ*=*π*_*T*_−*θπ*_*R*_−(1−*θ*)*π*_*P*_. The non-inferiority hypothesis (2.3) can be expressed as
2.4

The restricted maximum likelihood estimates (RMLEs) (denoted by ) of *π*_*T*_, *π*_*R*_ and *π*_*P*_ can be computed as follows. If the MLEs  of *π*_*T*_,*π*_*R*_,*π*_*P*_ satisfy the conditions:  and , we take ,  and ; otherwise, the RMLEs can be calculated by setting *π*_*T*_=*θπ*_*R*_+(1−*θ*)*π*_*P*_ in the likelihood function (2.1) and maximizing it with respect to *π*_*R*_ and *π*_*P*_. For the latter, it follows from Equation (2.1) that the RMLEs of *π*_*R*_ and *π*_*P*_ can be obtained by simultaneously solving the following equations in the parameter space *Θ*={(*π*_*P*_,*π*_*R*_):0≤*π*_*P*_<*π*_*R*_≤1}:


It is possible that there is no point (*π*_*P*_,*π*_*R*_) ∈*Θ* such that it satisfies the above equations, which implies that the likelihood function given in Equation (2.1) attains its maximum on the boundary of the parameter space *Θ*.

Following Tang and Tang [14], *ψ* can be estimated by , and its variance is given by , which can be estimated by , where  is some appropriate estimate of *π*=(*π*_*T*_,*π*_*R*_,*π*_*P*_), for example, taking  to be  or  which is the RMLE of *π*. Thus, the statistics for testing hypothesis (2.4) are given by


which are asymptotically distributed as the standard normal distribution under *H*_0_ as *n*_*T*_, *n*_*R*_ and *n*_*P*_ are sufficiently large. Hence, non-inferiority can be claimed if *T*_*W*_>*z*_1−*α*_ (or *T*_*R*_>*z*_1−*α*_), where *z*_1−*α*_ is the (1−*α*)-quantile of the standard normal distribution. When *π*_*P*_=0, *T*_*W*_ is the Wald-type statistic proposed in Blackwelder [20] and *T*_*R*_ is the test statistic given by Farrington and Manning [21] for two-arm noninferiority trials.

The signed root of the likelihood ratio statistic for testing hypothesis (2.4) is given by


which is asymptotically distributed as the standard normal distribution under *H*_0_ as *n*_*T*_, *n*_*R*_ and *n*_*P*_ are sufficiently large, where  with . Thus, non-inferiority can be claimed if *T*_*L*_>*z*_1−*α*_.

### *p*-value calculation methods

The non-inferiority hypothesis (2.2) can be claimed via the *p*-value method with the rule: *H*_0_ is rejected if the *p*-value is less than or equal to the prespecified significance level *α*. In what follows, we introduce five approaches for calculating *p*-values based on , which is the observed value of test statistic *T*_*j*_ (*j*=*W*,*R*,*L*) for the observed value  of (*X*_*T*_,*X*_*R*_,*X*_*P*_).

#### (1) Asymptotic method (AM)

It follows from the above arguments that all statistics *T*_*j*_’s (*j*=*W*,*R*,*L*) asymptotically follow the standard normal distribution under the null hypothesis *H*_0_:*ψ*≤0. Thus, the asymptotic *p*-value for testing hypothesis (2.2) via statistic *T*_*j*_ (*j*=*W*,*R*,*L*) based on  can be calculated by , where *Φ*(·) is the standard normal distribution function.

The above asymptotic approach for calculating *p*-value of testing hypothesis (2.2) via statistic *T*_*j*_ (*j*=*W*,*RW*,*L*) is established under the large sample theory. Its accuracy has long been suspected and criticized, especially when *n*_*T*_, *n*_*R*_ and/or *n*_*P*_ are small since the skewness of the underlying binomial distributions is not taken into consideration. Some higher order corrections such as the saddlepoint approximation [17] have been proposed to improve the accuracy of the normal approximation. In what follows, we will derive saddlepoint approximations to distributions of the three test statistics.

#### (2) Saddlepoint approximation method (SAM)

Since *X*_*T*_, *X*_*R*_ and *X*_*P*_ are independent and *X*_*i*_∼Bin(*n*_*i*_,*π*_*i*_) (*i*=*T*,*R*,*P*), the moment generating function of  is given by


with the cumulant generating function being


where −1≤*t*≤1. Thus, the first two derivatives of the cumulant generating function *K*(*t*) are given by


respectively. To obtain the saddlepoint approximation to , we need to solve the following saddlepoint equation:  whose unique solution is denoted as . Following Jing and Robinson [22], the saddlepoint approximation to the cumulative distribution function of statistic  is given by


where  and . Thus, the saddlepoint approximation to  (*j*=*W*,*R*,*L*) is given by


where  and ,  is the unique solution to equation:  for *j*=*W*,*R* with  and ,  and  with , and .

#### (3) Exact unconditional method (EUM)

When sample sizes (i.e., *n*_*T*_,*n*_*R*_,*n*_*P*_) are small, asymptotic methods may yield inflated type I error rates and their exact versions may provide reliable alternative. Under *H*_0_:*ψ*≤0 with *π*_*P*_<*π*_*R*_, parameters *π*_*R*_ and *π*_*P*_ must belong to the following constrained parameter space *Ω*={(*π*_*P*_,*π*_*R*_):0≤*π*_*P*_<*π*_*R*_≤1 if −*θπ*_*R*_<*ψ*<0, (−*ψ*−*θπ*_*R*_)/(1−*θ*)≤*π*_*P*_<*π*_*R*_<1 if −*π*_*R*_<*ψ*≤−*θπ*_*R*_, and empty set otherwise }. Under the null hypothesis, the probability density function (2.1) can be reexpressed by *π*_*T*_=*ψ*+*θπ*_*R*_+(1−*θ*)*π*_*P*_ with *π*_*R*_,*π*_*P*_ and *ψ* being nuisance parameters. These nuisance parameters can be eliminated by maximizing the null likelihood over the complete domain *Ω*. Similar to Tang and Tang [18], the exact unconditional *p*-value for testing *H*_0_:*ψ*≤0 via statistic *T*_*j*_ (*j*=*W*,*R*,*L*) based on  is defined as


where


and  is 1 if  and 0 otherwise.

#### (4) Approximate unconditional method (AUM)

According to Tang and Tang [18] and Tang, Tang and Rosner [23], the exact unconditional test is always conservative, i.e., its corresponding type I error rate is always less than or equal to the prespecified significance level. Following Tang and Tang [18], these nuisance parameters can be eliminated by evaluating their values at their corresponding RMLEs under *ψ*=0. The approximate unconditional *p*-value for testing *H*_0_:*ψ*≤0 via statistic *T*_*j*_ (*j*=*W*,*R*,*L*) based on  can be defined as .

#### (5) Bootstrap-resampling method (BTM)

Hypothesis testing based on the bootstrap-resampling method is usually recommended when sample sizes (i.e., *n*_*T*_, *n*_*R*_ and *n*_*P*_) are small [24] or data structure is sparse (e.g., *x*_*T*_ or *x*_*R*_ or *x*_*P*_ is close to zero or *n*_*T*_, *n*_*R*_ and *n*_*P*_, respectively). Given the observation , we compute the RMLEs  and  of parameters *π*_*T*_,*π*_*R*_ and *π*_*P*_, and calculate the observed value  of statistic *T*_*j*_ (*j*=*W*,*R*,*L*). Based on the RMLEs  and , we generate *B* bootstrap samples  from the following distribution:  for *k*=*T*,*R* and *P*. For each of the *B* bootstrap samples, we compute the observed value  of statistic *T*_*j*_ (*j*=*W*,*R*,*L*). Hence, an approximate *p*-value for testing *H*_0_:*ψ*≤0 via statistic *T*_*j*_ based on  is given by .

For any given observation , test statistic *T*_*j*_ (*j*=*W*,*R*,*L*) and *p*-value calculation method, we reject the null hypothesis *H*_0_ at the significance level *α* if  for *k*=AM, SA, EU, AU and BT.

### Simulation study

Simulation studies are conducted to investigate the performance of various test statistics together with the five *p*-value calculation methods in small-sample designs (e.g., *n*=30 and 60, where *n*=*n*_*P*_+*n*_*R*_+*n*_*T*_ with the allocation ratios *λ*_*P*_: *λ*_*R*_: *λ*_*T*_=1: *n*_*R*_/*n*_*P*_: *n*_*T*_/*n*_*P*_ taking to be 1:1:1, 1:2:2 and 1:2:3) in terms of type I error rate and power. For each (*n*_*P*_,*n*_*R*_,*n*_*T*_), we consider the following probability settings [19]: *π*_*P*_=0.05,0.10,0.15,…,0.50, *π*_*R*_=*π*_*P*_+0.05,*π*_*P*_+0.10,…,0.95, and *π*_*T*_=*θπ*_*R*_+(1−*θ*)*π*_*P*_, which corresponds to a total of 11,340 configurations of (*π*_*P*_,*π*_*R*_,*π*_*T*_), and the following two non-inferiority margins: *θ*=0.6 and 0.8. The nominal level is taken to be *α*=0.05. For the given values of *n* and allocation ratio *λ*_*P*_: *λ*_*R*_: *λ*_*T*_, *n*_*k*_ is given by *n*_*ℓ*_=*nλ*_*k*_/(*λ*_*P*_+*λ*_*R*_+*λ*_*T*_) for *ℓ*=*P*,*R* and *T*. Thus, given *n*, allocation ratio and (*π*_*P*_,*π*_*R*_,*π*_*T*_), the type I error rate for testing hypothesis *H*_0_:*ψ*≤0 versus *H*_1_:*ψ*>0 via test statistic *T*_*j*_ (*j*=*W*,*R*,*L*) at the significance level *α* is calculated by


for *k*=*AM*,*SAM*,*EUM*,*AUM* and *BTM*, whilst the corresponding power can be evaluated by replacing *H*_0_ in  by *H*_1_.

## Results

### Simulation study

**Figure 1:**
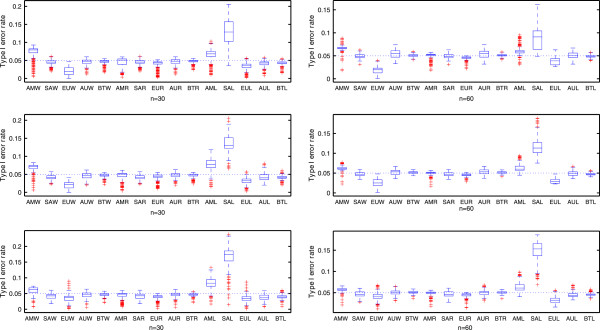
**Boxplots of the type I error rates of various test procedures together with three statistics when testing the non-inferiority hypothesis (2** ***.*** **2) at** ***α*** **=0** ***.*** **05.** AMk, SAk, EUk, AUk and BTk represent the AM, SA, EU, AU and BT test procedures with test statistic *T*_*k*_ for *k* = W, R and T, respectively.

To compare the performance of AM, SAM, EUM, AUM and BTM together with test statistics *T*_*W*_, *T*_*R*_ and *T*_*L*_ under the balanced and unbalanced designs, Figure 1 presents boxplots of their corresponding type I error rates for *n*=30 and 60, and *λ*_*P*_: *λ*_*R*_: *λ*_*T*_=1:1:1, 1:2:2 and 1:2:3, where AMk, SAk, EUk, AUk and BTk represent AM, SAM, EUM, AUM and BTM for test statistic *T*_k_ with k=W, R and L, respectively. Here, each boxplot in Figure 1 contains 2 (i.e., the number of non-inferiority margins) ×11,340 (i.e., the number of configurations for (*π*_*P*_,*π*_*R*_,*π*_*T*_))=22,680 data points. From Figure 1, we have the following findings. First, the medians of the type I error rates based on AUM and BTM are closer to the prespecified nominal level *α*=0.05 than those based on the other three *p*-value calculation methods for all three test statistics under consideration. Second, for AUM and BTM, the medians of the type I error rates for test statistics *T*_*W*_ and *T*_*R*_, which are 0.0495 and 0.0501 for AUM and 0.0494 and 0.0494 for BTM respectively, are closer to *α*=0.05 than those for test statistic *T*_*L*_, which are 0.0442 for AUM and 0.0442 for BTM. Third, for AM, SAM and EUM, their corresponding medians of type I error rates are 0.0649, 0.0455 and 0.0260 for test statistic *T*_*W*_, 0.0504, 0.0455 and 0.0488 for test statistic *T*_*R*_, and 0.0663, 0.1285 and 0.0332 for test statistic *T*_*L*_, respectively, which indicate that (i) the AM is liberal for test statistics *T*_*W*_ and *T*_*L*_, whilst it is valid for test statistic *T*_*R*_; (ii) the SAM can improve the accuracy of the normal approximation for test statistics *T*_*W*_ and *T*_*R*_; and (iii) the EUM is conservative for all test statistics. Fourth, the proportions of configurations whose type I error rates lie in the interval (0.045,0.055) for AM, SAM, EUM, AUM and BTM are 0.0747, 0.4691, 0.0710, 0.5154 and 0.7994 for *T*_*W*_, 0.5605, 0.4605, 0.4753, 0.7167 and 0.8370 for *T*_*R*_, and 0.0784, 0.0800, 0.0691, 0.4056 and 0.4889 for *T*_*L*_, respectively, which show that (i) AUM and BTM outperform the other three *p*-value calculation procedures, and (ii) *T*_*R*_ behaves better than the other two test statistics regardless of *p*-value calculation procedures. Fifth, the median of the type I error rates becomes more close to the prespecified nominal level as the total sample size *n* increases, whilst at the same time the variability of the type I error rates decreases. Sixth, the variability of the type I error rates for unbalanced designs is not significantly different from that for the balanced designs.

**Figure 2:**
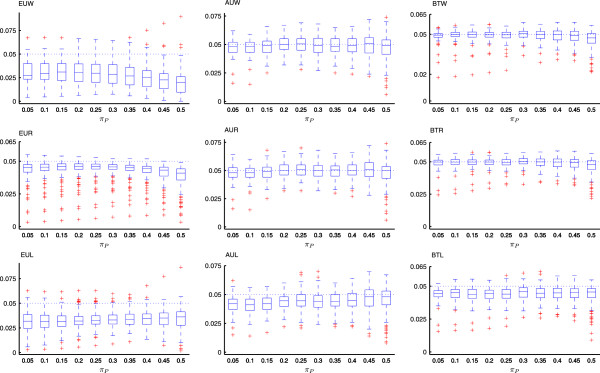
**Boxplots of the type I error rates of various test procedures together with three statistics against** ***π***_***P***_ **when testing the non-inferiority hypothesis (2** ***.*** **2) at** ***α*** **=0** ***.*** **05.** EUk, AUk and BTk represent the EU, AU and BT test procedures with statistic *T*_k_ for k = W,R and T, respectively.

To investigate the sensitivity of various *p*-value calculation procedures (i.e., AM, SAM, EUM, AUM and BTM) to different test statistics, Figure 2 presents boxplots of their corresponding type I error rates against *π*_*P*_ for test statistics *T*_*W*_, *T*_*R*_ and *T*_*L*_. Examination of Figure 2 shows that there is no significant effect of *π*_*P*_ on the type I error rate.

We also calculate powers of the five *p*-value calculation procedures together with the three test statistics at the nominal level *α*=0.05 when *π*_*T*_=*π*_*R*_ and *θ*=0.6 with the following settings: *n*=30 and 60, *π*_*P*_=0.15 and 0.3, and *π*_*R*_=0.5,0.8 and 0.95 for the balanced allocation 1:1:1 and unbalanced allocation 1:2:3. Results are reported in Table 1. Examination of Table 1 indicates that (i) *T*_*R*_ is generally more powerful than *T*_*W*_ and *T*_*L*_ for the EUM except for *π*_*R*_=0.95 with the unbalanced designs, (ii) *T*_*W*_ and *T*_*R*_ have similar powers for AM, AUM and BTM under our considered settings, (iii) a slight power difference is observed between *T*_*R*_ and *T*_*L*_ for AUM and BTM, (iv) there is slight power difference between balanced and unbalanced designs, and (v) power increases as *n* increases regardless of *p*-value calculation procedures or test statistics. Hence, we would recommend both AUM and BTM with *T*_*R*_ for hypothesis testing.

**Table 1:** **Exact powers (** ***%*** **) of various test procedures together with three statistics when** ***π***_***T***_ **=** ***π***_***R***_ **with** ***n*** **=30 and 60,** ***θ*** **=0** ***.*** **6and** ***α*** **=0** ***.*** **05**

				AM			SAM			EUM			AUM			BTM		
***n***	***λ***_***P***_: ***λ***_***R***_: ***λ***_***T***_	***π***_***P***_	***π***_***R***_	***T***_***W***_	***T***_***R***_	***T***_***L***_	***T***_***W***_	***T***_***R***_	***T***_***L***_	***T***_***W***_	***T***_***R***_	***T***_***L***_	***T***_***W***_	***T***_***R***_	***T***_***L***_	***T***_***W***_	***T***_***R***_	***T***_***L***_
30	1:1:1	0.15	0.5	13.4	12.3	13.9	43.6	21.2	22.5	5.0	18.1	15.3	18.2	18.2	14.9	17.9	18.0	16.2
			0.8	44.2	43.9	38.0	42.6	72.4	30.0	28.1	43.5	40.3	42.1	42.1	39.7	43.7	43.9	42.1
			0.95	86.0	85.8	75.2	95.6	97.7	36.8	67.8	79.1	76.0	74.2	74.2	71.3	75.8	75.4	75.0
		0.3	0.5	8.8	7.5	8.3	39.9	23.2	14.8	2.9	10.7	8.5	10.8	10.8	9.3	11.1	11.1	9.1
			0.8	30.1	29.2	22.0	21.1	35.6	26.6	15.9	29.2	24.8	30.0	30.0	26.8	30.2	30.7	29.0
			0.95	66.3	64.1	45.6	80.6	88.3	26.4	42.5	52.7	49.8	59.4	59.4	57.1	60.0	59.7	59.4
	1:2:3	0.15	0.5	14.2	11.9	18.6	33.2	28.2	24.7	13.2	17.9	13.7	21.7	21.7	19.7	20.3	20.0	18.4
			0.8	43.6	41.4	48.3	58.9	81.3	29.0	43.4	45.4	36.3	53.1	52.3	44.8	51.5	51.1	44.4
			0.95	83.0	82.9	83.9	97.1	98.8	36.0	85.6	79.6	82.6	85.9	84.3	79.9	85.4	84.9	80.6
		0.3	0.5	8.6	7.2	10.3	36.2	25.0	18.7	7.9	10.8	7.6	12.4	12.2	10.5	11.9	11.7	9.8
			0.8	29.7	27.4	30.1	26.1	57.9	25.9	28.9	31.9	22.7	35.0	33.6	28.2	34.4	33.7	29.5
			0.95	62.4	61.8	62.8	85.5	95.3	36.9	66.4	63.0	60.9	65.8	64.5	62.3	66.3	65.9	64.0
60	1:1:1	0.15	0.5	19.0	19.0	18.4	33.7	47.5	24.3	10.7	11.3	14.2	29.3	29.4	28.3	28.0	28.1	27.2
			0.8	65.8	67.8	59.6	85.2	92.4	38.9	55.0	56.3	48.7	71.4	71.4	71.4	71.1	71.1	70.7
			0.95	97.9	98.6	96.6	96.6	96.7	50.3	95.9	96.7	89.3	97.7	97.7	97.7	97.7	97.7	97.7
		0.3	0.5	9.7	9.4	9.5	43.2	21.5	16.9	4.7	5.3	4.2	17.0	17.2	15.3	14.1	14.3	13.1
			0.8	46.5	47.1	39.8	54.6	79.8	33.2	35.4	36.9	37.1	50.9	50.9	50.8	49.7	50.3	50.0
			0.95	91.3	93.3	85.7	95.3	96.0	47.0	85.9	87.8	71.3	88.0	88.0	88.0	89.6	89.3	89.5
	1:2:3	0.15	0.5	20.5	20.2	22.2	29.6	53.8	27.9	24.1	22.1	24.2	31.0	30.8	28.3	31.7	31.1	28.2
			0.8	72.5	72.5	69.1	92.3	96.4	40.9	73.9	73.3	79.2	77.0	76.9	75.9	78.3	78.1	76.7
			0.95	98.6	98.6	98.0	99.9	99.9	50.6	98.6	98.9	92.4	99.1	99.0	99.0	98.5	98.5	98.4
		0.3	0.5	10.3	10.0	10.3	42.9	25.0	20.1	12.3	10.3	10.1	15.8	15.7	13.5	15.8	15.4	13.4
			0.8	49.3	49.2	45.1	64.6	84.1	36.2	52.0	48.4	38.0	52.0	52.0	51.0	55.5	55.4	54.1
			0.95	90.4	90.3	88.7	99.1	99.4	51.3	90.9	92.5	82.4	92.1	92.0	92.0	91.8	91.7	91.7

### Real data example

An example from a pharmacological study of patients with functional dyspepsia (FD) and a placebo-controlled trail of subjects with acute migraine is used to illustrate our proposed methodologies. This example has been analyzed by Holtmann et al. [25] and Tang and Tang [14]. In this example, cisapride and simethicone can be regarded as the existing reference and new experimental treatments, respectively. In that study, among *n*=178 patients of FD, *n*_*P*_=61, *n*_*R*_=59 and *n*_*T*_=58 were randomized and treated in a doubly dummy technique with placebo, cisapride and simethicone, respectively; adverse events (e.g., diarrhea and pain) were happened in *x*_*P*_=7, *x*_*R*_=10 and *x*_*T*_=12 patients treated with placebo, cisapride and simethicone, respectively. It is of interest to test if simethicone is not inferior to cisapride in terms of rate of reporting adverse event in the presence of placebo. Given *θ*=0.6 and 0.8, the corresponding *p*-values for testing *H*_0_:(*π*_*T*_−*π*_*P*_)/(*π*_*R*_−*π*_*P*_)≤*θ* versus *H*_1_:(*π*_*T*_−*π*_*P*_)/(*π*_*R*_−*π*_*P*_)>*θ* based on the five *p*-value calculation procedures and three test statistics are reported in Table 2. By Table 2, there is no evidence to show that simethicone is noninferior to cisapride in the presence of placebo at the nominal level *α*=0.05, which is consistent with that given in Tang and Tang [14].

**Table 2:** **Various** ***p*** **-values for the pharmacological data set at the nominal level** ***α*** **=5** ***%***

	***θ***=0***.***6				***θ***=0***.***8		
Test method	***T***_***W***_	***T***_***R***_	***T***_***L***_		***T***_***W***_	***T***_***R***_	***T***_***L***_
AM	0.173	0.162	0.164		0.234	0.229	0.230
SAM	0.494	0.494	0.140		0.497	0.497	0.162
EUM	0.185	0.181	0.192		0.233	0.202	0.210
AUM	0.166	0.165	0.186		0.232	0.230	0.249
BTM	0.504	0.502	0.519		0.516	0.514	0.530

## Discussion

Simulation results demonstrate that our proposed score test statistic outperforms other test statistics in terms of type I error rate and power under our considered settings. The approximate unconditional and bootstrap-resampling methods perform better than other *p*-value calculation procedures in the sense that their corresponding type I error rates are closer to the prespecified nominal level and their corresponding powers are larger than those of other *p*-value calculation procedures. The exact unconditional method is conservative and time-consuming when sample sizes are large (e.g., see the 6th column in Table 3). The asymptotic tests are liberal since their type I error rates are greater than the prespecified nominal level *α*=0.05 in most cases. Comparing the approximate and exact unconditional methods, the approximate unconditional method provides a good alternative to the exact unconditional method in terms of computing time (e.g., see the 6th and 7th columns in Table 3) and type I error rate when sample sizes are large. In contrast, the computing burden of the bootstrap-resampling method is heavier than that of the approximate unconditional method (e.g., see the last two columns in Table 3).

**Table 3:** **Computing time (minutes) of the Type I error rates for 11340 configurations of (** ***π***_***P***_ **,** ***π***_***R***_ **,** ***π***_***T***_ **) together with three test statistics under five test methods**

***λ***_***P***_:***λ***_***R***_:***λ***_***T***_	***θ***	n	AM	SAM	EUM	AUM	BTM
1:2:3	0.6	30	3.3	269	2920	55.75	11700
		60	3.8	356	130950	357.3	20700

In this article, we concentrate on a three-arm non-inferiority trial with binary endpoints in which the marginal is defined as a fraction of the unknown difference in response probabilities between reference and placebo. The corresponding hypothesis (i.e.,  or *H*_0_:*π*_*T*_−*θπ*_*R*_−(1−*θ*)*π*_*P*_≤0) is considered since it is simple and only one single hypothesis is involved (e.g., see [6, 9, 14]). However, three-arm non-inferiority hypotheses with the marginal defined as the prespecified difference between treatments have received a considerable attention in recent years (e.g., see [5, 7]). They can be generally classified as the union type hypotheses (i.e., *H*_*U*0_: *π*_*R*_≥*h*_*P*_(*π*_*P*_) or *π*_*R*_≥*h*_*T*_(*π*_*T*_)) or the intersection type hypotheses (i.e., *H*_*U*0_: *π*_*R*_≥*h*_*P*_(*π*_*P*_) and *π*_*R*_≥*h*_*T*_(*π*_*T*_)), where *h*_*P*_(.) and *h*_*T*_(.) are any functions [15]. For specific choices of *h*_*P*_(.) and *h*_*R*_(.), this includes, for examples, hypotheses on the differences, the relative risks or the odds ratio of the proportions. While the union type hypotheses are suitable for showing both the superiority of the standard treatment as compared to placebo and the inferiority of the test treatment as compared to the standard treatment, the intersection type hypotheses are suitable for showing the test treatment is as effective as the standard or placebo treatments. We are working on statistical inference on a three-arm non-inferiority trial with the margin being a prespecifided difference between treatments when the primary endpoints are binary.

## Conclusions

According to the aforementioned observations, we can draw the following conclusions. In terms of type I error rates and powers, the approximate unconditional and bootstrap-resampling methods with score test statistic are recommended for hypothesis testing purpose when sample sizes are small in a three-arm non-inferiority trial. In terms of time-consuming and type I error rates and powers, the approximate unconditional method with score test statistic behaves the best among our considered *p*-value calculation procedures and test statistics.
